# Successful treatment of acute renal failure secondary to complicated infective endocarditis by peritoneal dialysis: a case report

**DOI:** 10.1186/s13104-017-2773-8

**Published:** 2017-09-07

**Authors:** Aisha M. Al-Osail, Ibrahim M. Al-Zahrani, Abdullah A. Al-Abdulwahab, Sarah M. Alhajri, Emad M. Al-Osail, Abdullah K. Al-Hwiesh, Fahad A. Al-Muhanna

**Affiliations:** 1 Department of Internal Medicine, The University of Dammam, Prince Saud bin Fahd Street, PO Box 3669, Khobar, 31952 Saudi Arabia; 2 Department of Internal Medicine, University of Dammam, PO Box 2435, Dammam, 31451 Saudi Arabia; 3 Nephrology Division, Department of Internal Medicine, University of Dammam, PO Box 2435, Dammam, 31451 Saudi Arabia

**Keywords:** Infective endocarditis, *Staphylococcus aureus*, Automated peritoneal dialysis, Acute kidney injury

## Abstract

**Background:**

Infective endocarditis is one of the most common infections among intravenous drug addicts. Its complications can affect many systems, and these can include acute renal failure. There is a scarcity of cases in the literature related to acute renal failure secondary to infective endocarditis treated with peritoneal dialysis. In this paper, the case of a 48-year-old Saudi male is reported, who presented with features suggestive of infective endocarditis and who developed acute kidney injury that was treated successfully with high tidal volume automated peritoneal dialysis. To our knowledge, this is the second report of such an association in the literature.

**Case presentation:**

A 48-year-old Saudi gentleman diagnosed to have a glucose-6-phosphate dehydrogenase deficiency and hepatitis C infection for the last 9 years, presented to the emergency department with a history of fever of 2 days’ duration. On examination: his temperature = 41 °C, there was clubbing of the fingers bilaterally and a pansystolic murmur in the left parasternal area. The results of the blood cultures and echocardiogram were supportive of the diagnosis of infective endocarditis, and the patient subsequently developed acute kidney injury, and his creatinine reached 5.2 mg/dl, a level for which dialysis is essential for the patient to survive.

**Conclusion:**

High tidal volume automated peritoneal dialysis is highly effective as a renal replacement therapy in acute renal failure secondary to infective endocarditis if no contraindication is present.

## Background

Drug addiction is one of the principal economic problems not only in the Gulf region and the Middle East but worldwide because of its associated complications [1–4]. Infective endocarditis is a common infection that occurs in intravenous drug addicts (IVDAs) and is commonly caused by the *Staphylococcus aureus* organism, often affecting the tricuspid valve; however, the involvement of other valves and organisms is not uncommon [5, 6]. Complications following infective endocarditis are varied and can involve many systems at the same time. Such complications include septic emboli, stroke and renal complications, including immune complex glomerulonephritis, antibiotic-induced interstitial nephritis, acute tubular necrosis and renal infarction [7–16]. The usual management of acute renal failure is intermittent haemodialysis (HD) or continuous renal replacement therapy. Although automated peritoneal dialysis (APD) therapy is also considered for treatment of acute renal failure, there are few reports of the use of this method for renal failure secondary to infective endocarditis. High tidal volume APD can also be effective as a renal replacement therapy in acute kidney injury (AKI) secondary to infective endocarditis provided no contraindication is present.

### Ethical considerations

Written informed consent was obtained from the patient for publication of this Case Report and any accompanying images. A copy of the written consent is available for review by the Editor-in-Chief of this journal.

## Case presentation

A 48-year-old Saudi gentleman with a known history of IV drug abuse and who had had a hepatitis C infection for the last 9 years presented to the emergency department with a fever of 2 days’ duration. He had been an IV drug user (heroin) for 10 years. He presented to the emergency department with a high grade, continuous fever that was not relieved by antipyretics, and which was accompanied by chills and sweating. He denied any history of upper respiratory tract infection, shortness of breath, chest pain, palpitations, abdominal pain, nausea/vomiting, changes in bowel habit, headache, neck stiffness, photophobia, dysuria or frequent urination. There was no history of recent travel, animal exposure, contact with an ill person or a patient with tuberculosis, ingestion of raw milk, skin rash, oral ulcers, dry mouth or eyes, or joint pain. In the physical examination, his vital signs were as follows: temperature = 41 °C, blood pressure = 144/60 mmHg, pulse = 125 beats/min, respiratory rate = 20 breaths/min, oxygen saturation = 97% in room air. Hands and arms: Clubbing grade III, multiple IV punctures, no splinter haemorrhage or Osler’s nodules, no Janeway lesions. Head and neck: Normal. Chest: Vesicular breathing and no added sounds. Heart: Soft S1, pansystolic murmur at left parasternal area, grade III increase with inspiration and hand grip, no pericardial rub. Abdomen: Normal. Lower Limbs: Clubbing grade III, multiple IV punctures. Neurological examination: Conscious, oriented to time, person and place, normal motor, sensory and cerebellar examination. Blood tests showed the following: WBC = 17.8, neutrophils = 79%, lymphocytes = 5%, band = 9%, monocyte = 6%, eosinophil = 1%, haemoglobin = 10.9 g/l, MCV = 86.6, MCH = 26.8, MCHC = 31, platelets = 332, ESR = 106 mm/h. Lactic acid was high (5.3 mmol/l) and the initial renal function and liver function tests were normal. Three separate blood cultures were drawn from different sites over a 1-h period before starting the antibiotics and on the subsequent day a positive result for methicillin-sensitive *S. aureus* (MSSA) was revealed. The echocardiogram showed small vegetation over the anterior chordae tendineae of the anterior mitral valve, and there was no evidence of intramyocardial abscess or fistula. The patient was started on vancomycin and gentamicin until the final culture results were available. Vancomycin was then stopped and cloxacillin was started. Gentamicin administration was maintained for 5 days for a synergistic effect. On the seventh day following admission, the patient developed progressive AKI. His renal function tests deteriorated gradually, with the BUN level reaching 67 mg/dl, and the creatinine level was 5.2 mg/dl. Urine analysis was normal and negative for eosinophils, and the kidney ultrasound showed a normal size, shape and position of the kidneys, and the renal arteries were normal. The nephrology team evaluated the patient and decided to commence high tidal volume APD with 30 l of Physioneal over 24 h, each refill was 1.5 l. Gentamicin was stopped after the patient completed a 1-week course. The gentamicin trough level was 0.2 μg/ml (normal level is <0.2 μg/ml), whereas the peak level was 3.5 μg/ml (normal level is 4–8 μg/ml). The patient showed clinical and laboratory improvement, see results in Table 1. He required APD for only 1 week. A 4-week course of cloxacillin was completed and the patient was discharged in good condition. The patient was referred to a rehabilitation centre for drug addiction. We followed up with renal function testing for 1 year, and all results were normal. The final results are listed in Table 1.

**Table 1:** Renal and biochemical profile of the patient before and after APD

Day	BUN (mg/dl)	Creatinine (mg/dl)	Na	K	Cl	CO_2_
Day of admission	16	0.9	138	3.9	99	28
7th day following admission	31	2.1	133	3.7	96	21
8th day following admission	43	3.4	132	4	100	20
11th day following admission	63	4.7	132	4.9	102	19
Day of tidal APD (12th day)	67	5.2	132	5.2	99	14
*13th day*	*50*	*3.5*	*134*	*3.6*	*96*	*20*
*14th day*	*30*	*2.1*	*135*	*3.5*	*99*	*26*
*15th day*	*17*	*1.0*	*133*	*3.2*	*99*	*28*
After 1 year	16	0.9	137	3.9	102	25

The values in italics is after peritoneal dialysis

## Discussion

Infective endocarditis is one of the most common infections among IVDAs and can affect any valve, including the tricuspid (60–70%), mitral and aortic valves (20–30%), and the pulmonary valve (<1%). Multiple valves can be affected at the same time, as reported with drug abusers. Recent studies on valve involvement in drug abusers have reported that whereas left-side valves are more commonly affected in the general population, the right-side valves are more commonly affected in IVDAs worldwide, with opposite results found in the Middle East [1, 2, 17, 18]. In this patient, it can be seen that the diagnosis of infective endocarditis is, without a doubt, correct. He showed the two major Duke criteria of (1) positive blood culture, and (2) a new murmur and vegetation seen through the echocardiogram. The most common organism reported in the literature is *S. aureus*, which is found in 50–90% of patients with infective endocarditis [19, 20]. Complications from infective endocarditis can be considered multisystem, with one of the most important affected systems being the renal system, as endocarditis itself can be manifested in acute interstitial nephritis, focal and/or diffuse proliferative glomerulonephritis, renal cortical necrosis or antibiotic-related interstitial nephritis [7, 21–23]. This patient developed AKI, which could either have been because of the infective endocarditis itself or antibiotic related. No renal biopsy was performed to confirm this. Haemodialysis is one of the known risk factors for infective endocarditis, as has been shown in several published studies. One of the most important of these studies was the International Collaboration on Endocarditis-Prospective Cohort Study (ICE-PCS), which concluded that of all cases of infective endocarditis (bacterial in origin), 21% in North America were among HD patients [24, 25]. No cases have been documented in peritoneal dialysis (PD) patients. PD for AKI patients still constitutes the mainstay of therapy in many developing countries because of its availability and ease of administration. Although its safety is being confirmed, PD is used less and less in patients with AKI, and this might be because of lack of experience, lack of facilities or both. In the PD centre at the King Fahd Hospital of the University, a new technique in PD catheter insertion has been developed that made the procedure much simpler, safer and more efficient than before [26]. By using this technique, it is believed that PD should not be discarded as a worthwhile therapeutic option for a patient with AKI because of its technical simplicity, excellent cardiovascular tolerance, absence of an extracorporeal circuit, lack of bleeding risk and low risk of hydro-electrolyte imbalances. In general, the indication for initiation of dialysis includes acidaemia—life-threatening and causing hemodynamic instability—life-threatening hyperkalaemia, volume overload unresponsive to diuretics and uraemia, including pericarditis and encephalopathy [27]. In tidal PD (TPD), only a portion of the dialysate is drained from the peritoneal cavity after the initial PD fluid exchange. The drained volume is replaced by fresh dialysate (the tidal volume), with each cycle leaving a variable amount of dialysate (residual volume) in constant contact with the peritoneal membrane until the end of the dialysis session [28]. There are several advantages of PD in comparison to other renal replacement therapies in treating AKI. No systemic anticoagulation is required, and this is particularly important in AKI following major surgery or when haemorrhaging or coagulation disorders are present. The use of anticoagulation with heparin or low molecular weight heparin in HD or continuous renal replacement theory (CRRT) would increase the risk of bleeding. With PD, no systemic anticoagulation is required, and thus the bleeding risk would not be increased. In cases where intraperitoneal heparin is needed, heparin is not effectively absorbed through the peritoneum, and therefore, there should not be any significant systemic effect on coagulation. Patients on PD are haemodynamically more stable than those on HD. Many patients with AKI have hypotension and shock, and they tolerate HD or even CRRT poorly. In general, these patients tolerate PD better than HD or CRRT. Hypotension and arrhythmia are rarely induced by the PD treatment. The reasons include the following:

- Lack of extracorporeal circulation, causing immediate reduction of blood volume.
- Slower fluid removal from the blood volume, allowing more time for equilibrium between different fluid compartments to be reached.
- Slower fall of serum urea level, allowing time for equilibrium between intracellular and extracellular urea levels to be reached. This prevents the acute drop in plasma osmolality that occurs in HD as a result of rapid extracellular urea removal.
- Slower rate of change in electrolyte (potassium and calcium) levels.

In addition, PD may lead to more rapid, and a higher chance of, recovery of renal function. It is well documented that PD preserves residual renal function better than HD in patients with end-stage renal failure. Although the exact mechanism remains unknown, it is generally believed that this is related to the more stable haemodynamics with PD, and possibly is also a result of lower complement activation and higher middle molecule clearance with PD. The same may apply to PD for acute renal failure. In a retrospective study of 31 patients with AKI caused by malignant hypertension in the period of 1997–2000, 11 out of 20 patients who received PD became dialysis free, whereas none of the 11 patients who received HD had recovered their renal function. The patient described in this paper presented with features suggestive of infective endocarditis and developed AKI. The patient was treated successfully with high tidal volume APD, with full recovery of renal function. To our knowledge, this is the second report of such an association in the literature.

## Conclusion

High tidal volume APD can be effective as a renal replacement therapy in AKI secondary to infective endocarditis provided no contraindication is present.

## Abbreviations

AKI: acute kidney injury
APD: automated peritoneal dialysis
CAPD: continuous ambulatory peritoneal dialysis
G6PD: glucose-6-phosphate dehydrogenase
HD: haemodialysis
IE: infective endocarditis
IVDAs: intravenous drug addicts
PD: peritoneal dialysis
TPD: tidal peritoneal dialysis
